# 30-Day Survival Probabilities as a Quality Indicator for Norwegian Hospitals: Data Management and Analysis

**DOI:** 10.1371/journal.pone.0136547

**Published:** 2015-09-09

**Authors:** Sahar Hassani, Anja Schou Lindman, Doris Tove Kristoffersen, Oliver Tomic, Jon Helgeland

**Affiliations:** 1 Norwegian Knowledge Centre for the Health Services, Oslo, Norway; 2 Department of Medical Genetics, University of Oslo and Oslo University Hospital, Oslo, Norway; 3 NORMENT, KG Jebsen Centre for Psychosis Research, Oslo University Hospital, Oslo, Norway; Chinese Academy of Medical Sciences, CHINA

## Abstract

**Background:**

The Norwegian Knowledge Centre for the Health Services (NOKC) reports 30-day survival as a quality indicator for Norwegian hospitals. The indicators have been published annually since 2011 on the website of the Norwegian Directorate of Health (www.helsenorge.no), as part of the Norwegian Quality Indicator System authorized by the Ministry of Health. Openness regarding calculation of quality indicators is important, as it provides the opportunity to critically review and discuss the method. The purpose of this article is to describe the data collection, data pre-processing, and data analyses, as carried out by NOKC, for the calculation of 30-day *risk-adjusted survival probability* as a quality indicator.

**Methods and Findings:**

Three diagnosis-specific 30-day survival indicators (first time acute myocardial infarction (AMI), stroke and hip fracture) are estimated based on all-cause deaths, occurring in-hospital or out-of-hospital, within 30 days counting from the first day of hospitalization. Furthermore, a hospital-wide (i.e. overall) 30-day survival indicator is calculated. Patient administrative data from all Norwegian hospitals and information from the Norwegian Population Register are retrieved annually, and linked to datasets for previous years. The outcome (alive/death within 30 days) is attributed to every hospital by the fraction of time spent in each hospital. A logistic regression followed by a hierarchical Bayesian analysis is used for the estimation of *risk-adjusted survival probabilities*. A multiple testing procedure with a false discovery rate of 5% is used to identify hospitals, hospital trusts and regional health authorities with significantly higher/lower survival than the reference. In addition, estimated *risk-adjusted survival probabilities* are published per hospital, hospital trust and regional health authority. The variation in risk-adjusted survival probabilities across hospitals for AMI shows a decreasing trend over time: estimated survival probabilities for AMI in 2011 varied from 80.6% (in the hospital with lowest estimated survival) to 91.7% (in the hospital with highest estimated survival), whereas it ranged from 83.8% to 91.2% in 2013.

**Conclusions:**

Since 2011, several hospitals and hospital trusts have initiated quality improvement projects, and some of the hospitals have improved the survival over these years. Public reporting of survival/mortality indicators are increasingly being used as quality measures of health care systems. Openness regarding the methods used to calculate the indicators are important, as it provides the opportunity of critically reviewing and discussing the methods in the literature. In this way, the methods employed for establishing the indicators may be improved.

## Introduction

Several different quality indicators, with the aim of monitoring the quality of care in the health services, have been developed, studied and reported in the literature during the past decades [1–5]. Quality indicators are measured with the aim to provide information regarding how well a hospital performs its services [6]. However, such measurements alone does not assess the quality of care at a hospital [7]. By considering several indicators that describe the relevant aspects of a hospital, one may get a picture of the overall performance of the hospital.

Mortality within a fixed-time period (commonly 30 days) after admission is among the reported indicators and is considered to be an effective means for measuring the outcome of hospital care [8]. As mortality is perceived a negative framing, the Norwegian Knowledge Centre for the Health Services (NOKC) reports the estimated 30-day *survival probabilities* as routinely reported quality indicators for Norwegian hospitals [9–12]. This is in contrast to the vast majority of other quality indicator systems which report *mortality* or standardized mortality ratio (SMR).

Three disease-specific and one hospital-wide survival indicators are estimated. The disease-specific conditions are first time acute myocardial infarction (AMI), stroke and hip fracture. The hospital-wide survival is calculated for a subset of diagnoses, which accounts for approximately 80% of the total number of deaths within 30 days after admission to Norwegian hospitals. The patient administrative data used for calculation of the hospital-wide indicator correspond to the data commonly used for the hospital standardized mortality ratios.

The four quality indicators from NOKC are estimated and reported at hospital level, hospital trust level and regional health authority level. We employ information from all admissions for a patient when calculating the indicators i.e. for transferred patients, the outcome (of alive or death within 30 days) is attributed to every hospital by the fraction of time spent in them (weighted admissions) [13].

Several studies have highlighted the growing need for methodological peer-reviewed publications and guidelines that can be employed for studying and producing health care indicators [14, 15]. This technical article aims to give a detailed description of the methods we employed for data collection, data processing and statistical analysis of the 30-day survival indicators.

## Material and Methods

### Data

#### Data Sources

**Patient Administrative System (PAS) Data**

Each record in PAS data contains information from a single ward admission. The same patient might have several records as the patient is transferred between wards and even hospitals. PAS data comprise admission category (i.e. elective or emergency), diagnosis codes (both primary and secondary), codes for medical procedures, age, gender, date and time for ward admission/discharge.

PAS data are collected from two sources:

1. Data from 2009 and earlier: PAS data extracted directly from the hospitals using an in-house developed system for the purpose of quality monitoring [16]. At each hospital the same patient has a unique patient record key for the whole period.
2. Data from 2010 and onward: PAS data are provided by the Norwegian Patient Register (NPR); it is mandatory for all Norwegian hospitals to submit data to NPR. The records from each patient have the same unique patient record key throughout the dataset period.

**The National Registry**

Data from the National Registry are provided by Statistics Norway [17]. All permanent residents in Norway have a Personal Identification Number (PIN). Statistics Norway have yearly received files from the hospitals (for data up to the year 2009) or from NPR (for data for 2010 and onward), containing the PIN and the unique patient record key (mentioned above). An encrypted PIN is generated by Statistics Norway, enabling us to track all patients between hospitals and link the patients to their hospitalizations throughout the data collection period. Each year, updated information for the respective patients; age, gender, date of death (in-hospital and out-of-hospital) and patients’ status (e.g. habitant or emigrated) is retrieved from the National Registry.

#### Data Pre-processing

**Constructing episodes of care**

We define an episode of care as the continuous course of care of a patient. This may include a sequence from admission to a hospital, through within-hospital transfers and transfers between different hospitals, to the discharge of the patient.

**Fig 1:**
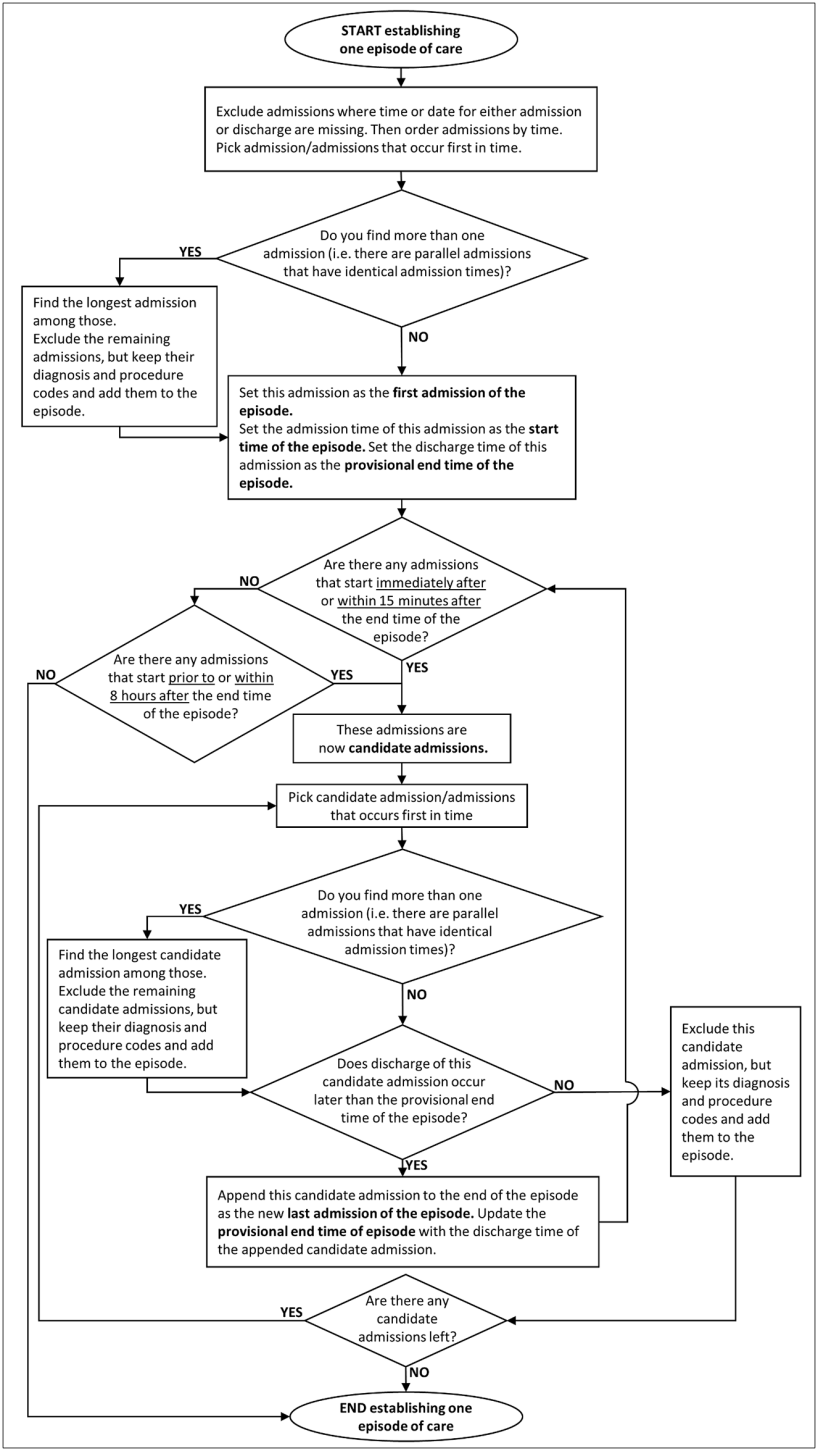
Flowchart of constructing one episode of care (for one patient). Square boxes contain the instructions, ovals represent the start and end point of the process and diamonds indicate decisions.

An overview of how the episodes of care are constructed is given in the following, while the detailed description of the algorithm is shown in a flowchart (see Fig 1).

- Firstly, ward admissions that lack time or date for admission or discharge are excluded.
- Thereafter, ward admissions are ordered in time sequence.
- Ward admissions are then aggregated into a single episode of care, if the time difference between the discharge of the first one and admission of the next one is less than eight hours.
- In case of parallel ward admissions:
  ○ If the parallel ward admissions start at the same time, the shorter ones are excluded, while their diagnosis and procedure codes are still taken into account.
  ○ If the parallel ward admissions start at different time points, the ones that start later are excluded, while their diagnosis and procedure codes are still taken into account.

**Fig 2:**
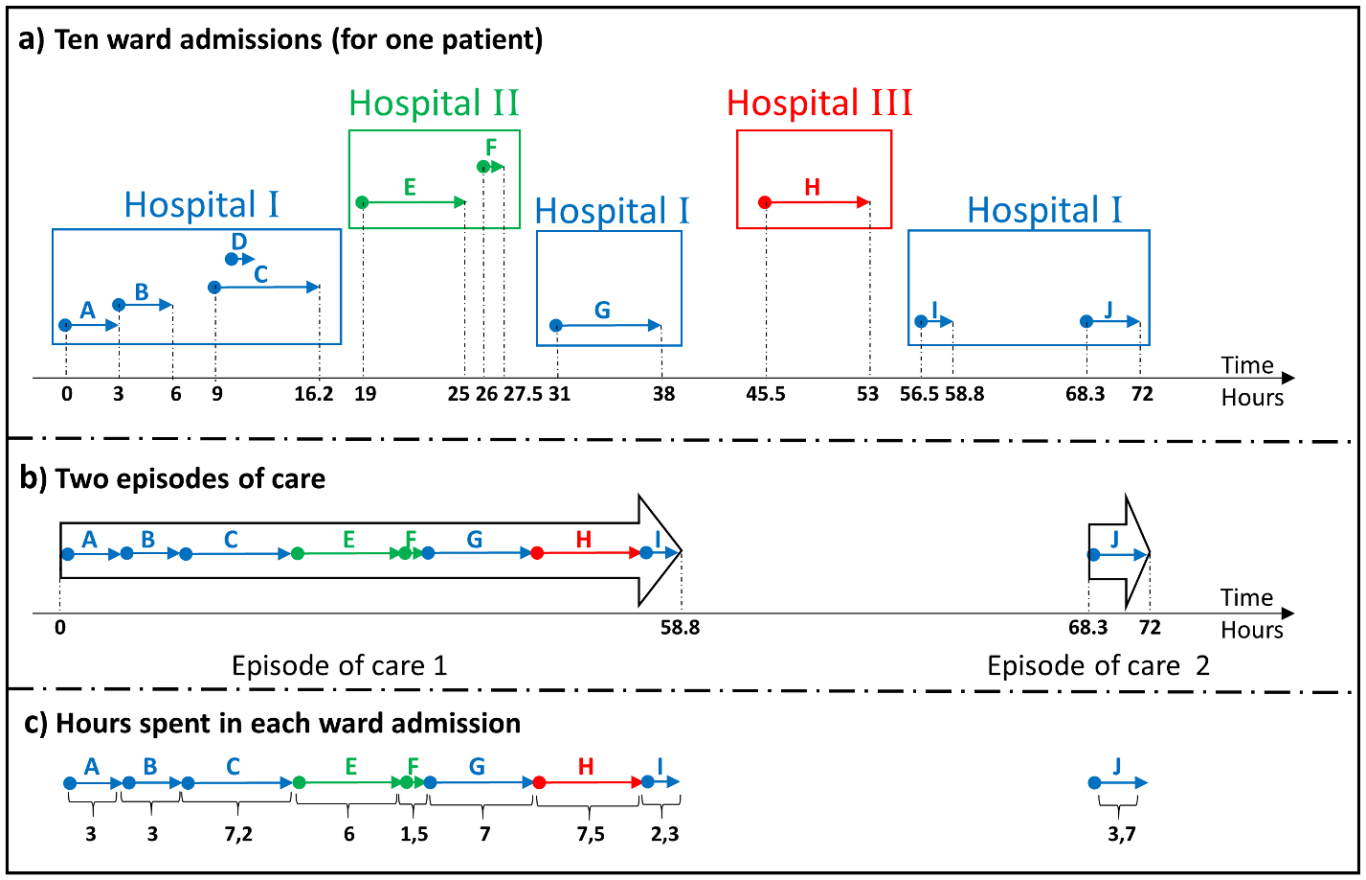
Constructing episodes of hospital cares and calculating the hospital weights. **(a)** Ten ward admissions are labelled with capital letters (from A to J) and are illustrated with arrows that are of three different colours for three different hospitals. The horizontal axis gives the time of admission/discharge (in hours) for every ward admission (for the sake of simplicity, the time of the first admission is assumed to be zero). **(b)** Two episodes of care, labelled with Episode 1 and 2 (illustrated with wide black arrows), are constructed from the ten ward admissions in a. **(c)** The length of stay (in hours) for each ward admission is shown.

Fig 2 illustrates an example of constructing episodes of care according to the above described rules. Ten ward admissions labelled from A to J are shown in Fig 2a. The patient is first admitted to Hospital I at time zero, and is then transferred within the same hospital (ward admissions A to D). Thereafter, the patient is transferred to Hospital II, and back to Hospital I, followed by a stay at Hospital III, and finally discharged from Hospital I. If the time difference is more than eight hours, a new episode of care is generated. The time difference between ward admissions I and J are more than eight hours, and a new episode of care is therefore generated. Two episodes of care are constructed for the patient based on these admissions (illustrated in Fig 2b).

**Calculating hospital weights**

In the analysis, episodes spanning more than one hospital must be attributed to these hospitals in a meaningful way. For this purpose, episodes are assigned hospital weights. For the episodes of care containing more than one hospital (i.e. for transferred patients), we establish the hospital weights proportional to the fraction of time (within the episode of care) spent in each hospital [13]. Weights are restricted to the interval [0.05, 1.00]. One single episode of care has a total weight of 1. For the episodes of care including only one hospital, the hospital weight will be 1.

The amount of hours spent in each hospital in the previous example (in Fig 2) are shown in Fig 2c and the weights assigned to each hospital are calculated below:

Weights calculated based on episode 1:

$$\text{Hospital I's weight}\;=\;\frac{\;Lengths\;of\;stay\;in\;A,\;B,\;C,\;G\;and\;I}{\text{Total length of stay }}\;=\;\frac{22.5}{37.5}\;=\;0.6$$

$$\text{Hospital II's weight}\;=\;\frac{\;Lengths\;of\;stay\;in\;E\;and\;F}{\text{Total length of stay }}\;=\;\frac{7.5}{37.5}\;=\;0.2$$

$$\text{Hospital III's weight}\;=\;\frac{\;Lengths\;of\;stay\;in\;H}{\text{Total length of stay }}\;=\;\frac{7.5}{37.5}\;=\;0.2$$

Weight calculated based on episode 2:

Hospital I's weight = 1

Hospital II's weight = 0

Hospital III's weight = 0

The total weight of a hospital is defined as the summed weights for all of the episodes of care belonging to that hospital.

Total weights calculated based on both episodes:

Hospital I's weight = 1.6

Hospital II's weight = 0.2

Hospital III's weight = 0.2

#### Case selection

The case selection criteria are different for the hospital-wide data set and the three diagnosis-specific data sets. Diagnosis codes are the ICD-10 diagnosis codes from the Norwegian version of ICD-10 [18]. In this technical article, however, the corresponding English description of the codes are taken from the original WHO (World Health Organisation) version of ICD-10 [19]. Moreover, wherever “x” is written in a diagnosis code, it may be replaced by nothing or anything i.e. an empty string or any number may replace “x”.

**Hospital-wide data set**

Different admissions may have different sets of diagnosis and procedure codes. The first non-vague primary diagnosis [20] is used to identify the patients in the hospital-wide data set, and is defined as the first occurring (according to the time sequence of admissions) primary diagnosis that is not on the list of vague diagnoses (see S1 Appendix). If no such diagnosis is found, the last occurring diagnosis is used.

Inclusion Criteria

Episodes of care are included if

1. The episode discharge date is between January 1 and December 31 of a given year i.e. a one-year period hospital-wide data set is selected.
2. The first non-vague primary diagnosis code belongs to a subset of Clinical Classifications Software (CCS) [21, 22] categories, accounting for approximately 80% (or 90%) of the total 30-day mortality in Norwegian hospitals (for more information about the CCS categories see S2 Appendix).
3. Admission date is recorded (not missing).
4. Admission category is recorded (not missing), i.e. elective or emergency.
5. The patient has a valid encrypted PIN and information regarding the patient’s status (alive or dead) 30 days from the admission date is recorded.

The admission date and admission category refer to the first ward admission in the episode of care.

Exclusion Criteria

Episodes of care are excluded if

1. A previous episode of care, whose first non-vague primary diagnosis code belongs to the Clinical Classifications Software (CCS) categories of interest, exists within 30 days prior to the admission date.
2. Diagnosis codes for palliative care (Z51.5x) is recorded either as primary or as secondary diagnosis.

**Diagnosis specific data sets**

Inclusion Criteria

Episodes of care are included if

1. The episode discharge date is between January 1 of a given year and December 31 in the three years that follow, i.e. three-year period diagnosis specific data sets are selected.
2. The diagnosis codes correspond to the criteria specified in Table 1. For transferred patients, the diagnoses must occur at the *first hospital* in the episode of care.
3. Admission date is recorded (not missing).
4. Admission category is recorded as emergency.
5. The patient has a valid encrypted PIN and information regarding the patient’s status (alive or dead) 30 days from the admission date is recorded.

**Table 1:** Diagnosis types and diagnosis codes^a^ for diagnosis specific data sets.

Indicator categories	ICD-10 diagnosis code	Description	Diagnosis Type
**AMI**	I21.0	Acute transmural myocardial infarction of anterior wall	Primary or Secondary
	I21.1	Acute transmural myocardial infarction of inferior wall	Primary or Secondary
	I21.2	Acute transmural myocardial infarction of other sites	Primary or Secondary
	I21.3	Acute transmural myocardial infarction of unspecified site	Primary or Secondary
	I21.4	Acute subendocardial myocardial infarction	Primary or Secondary
	I21.9	Acute myocardial infarction, unspecified	Primary or Secondary
**Hip fracture**	S72.0	Fracture of neck of femur	Primary or Secondary
	S72.1	Pertrochanteric fracture	Primary or Secondary
	S72.2	Subtrochanteric fracture	Primary or Secondary
**Stroke**	I61.x	Intracerebral haemorrhage	Primary
	I63.x	Cerebral infarction	Primary
	I64.x	Stroke, not specified as haemorrhage or infarction	Primary

^a^The diagnosis codes refer to any ward admission in the first hospital in the episode of care.

The admission date and admission category, however, refer to the first *ward admission* in the *episode of care*.

Exclusion Criteria

Episodes of care are excluded if the criteria specified in Table 2 are met.

**Table 2:** Exclusion criteria for diagnosis specific data sets.

Indicator categories	Age	Previous admission exist (within the same diagnosis category)
**AMI**	Age < 18 years	7 years prior to the current episode of care’s admission date
**Hip fracture**	Age < 18 years	60 days prior to the current episode of care’s admission date
**Stroke**	Age < 65 years	28 days prior to the current episode of care’s admission date

### Statistical Analysis

#### Definition

By the 30-day survival indicator, we refer to the risk-adjusted probability of survival within 30 days of the (episode) admission date.

#### Modelling

The survival probabilities are estimated in three steps:

**Step 1: Generalised Linear Model (GLM) Regression Analysis**

A GLM regression model is fitted to the set of episode data from all of the hospitals, where the explanatory variables are case-mix variables as well as hospital weights and (for the three-year diagnosis-specific data sets) a time trend, linear in admission year. The response variable is death within 30 days from the admission date.

The case-mix variables used for the hospital-wide and for the diagnosis specific data sets are slightly different and are, therefore, described separately in the following.

- Case-mix variables for hospital-wide data analysis:
  1. **Age** (years)
  2. **Gender**
  3. **Pre-admissions**: Number of hospital episodes during the previous two years
  4. **The Charlson Comorbidity Index (CCI)**: CCI, based on all diagnosis codes occurring in episodes from the last three years prior to, but not including, the current episode (revised ICD-10 implementation of Quan et al. [23, 24] is used)
  5. **Admission category:** Elective or emergency
  6. **Clinical Classifications Software (CCS) category**
- Case-mix variables for diagnosis-specific data analyses:
  1. **Age (years)**
  2. **Gender**
  3. **Pre-admissions**: Number of hospital episodes during the past two years
  4. **The Charlson Comorbidity Index (CCI)**: CCI, based on all diagnosis codes occurring in episodes from the last three years prior to, but not including, the current episode (revised ICD-10 implementation of Quan et al. [23, 24] is used)
  5. **The type of stroke (stroke data only):** Intracerebral haemorrhage, cerebral infarction and stroke, not specified as haemorrhage or infarction (ICD 10 codes: I61.x, I63.x and I64.x, respectively)

Age is modelled by natural splines [25] while pre-admissions and CCI are modelled by fractional polynomials [26].

**Step 2: Identifying outliers by Multiple Significance Testing**

The regression coefficient of each hospital is compared to a reference value. The reference value is the 10%- trimmed mean of the regression coefficients, i.e. the lowest 10% and the highest 10% of the regression coefficients are excluded prior to calculation of the mean value.

Multiple significance testing is then used to identify hospitals (hospital trusts or regional health authorities, depending on the level of the analysis) deviating from the reference (either lower or higher survival). Multiple testing is performed using the Benjamini-Hochberg method [27] with a False Discovery Rate (FDR) of 5%. Two-sided tests are used.

**Step 3: Survival Probability Calculation by Shrinkage Estimation**

The estimated regression coefficients for each hospital are shrunk towards the reference value by a Hierarchical Bayesian Model [28, 29]. The prior distribution is as follows: the true hospital regression coefficients are assumed to follow a Student’s t-distribution with five degrees of freedom, multiplied by a scale factor σ. The precision σ^-2^ has a gamma distribution with both shape and rate parameters equal to 0.01.

Hypothetical survival probabilities for each patient are thereafter calculated using the adjusted GLM with the shrunk hospital coefficients, assigning all patients to each of the hospitals in turn. The survival probabilities for each hospital are then computed as the mean survival probabilities for all the patients when they are assigned to each hospital.

#### Hospital and hospital trust inclusion/exclusion

Several Norwegian hospitals are small and have a limited number of relevant cases. We thus do not consider hospitals (or hospital trusts, depending on the level of the analysis) in the analysis for hospital-wide data, if their total weight (according to the section Calculating the hospital weights) calculated for the one-year period (for the patients belonging to the hospital-wide data), is less than 400.

Similarly, hospitals (or hospital trusts) are not considered in the analysis for diagnosis-specific data, if their weight calculated for the last year (for the patients belonging to the respective diagnosis-specific data) is less than 20 or their weight calculated for the whole three-year period (for the patients belonging to the respective diagnosis-specific data) is less than 100.

### Ethics

The Norwegian Data Inspectorate and the Ministry of Health approved the data collection for 30-day mortality. Ethical approval was not necessary according to the Regional Ethics Committee, as existing data were employed for quality improvement purposes.

The Norwegian Knowledge Centre for the Health Services (NOKC) receives an encrypted PIN, generated by Statistics Norway, which does not enable us to have access to patient identifying information. The Norwegian Knowledge Centre does not have any direct interaction with patients.

## Results

**Fig 3:**
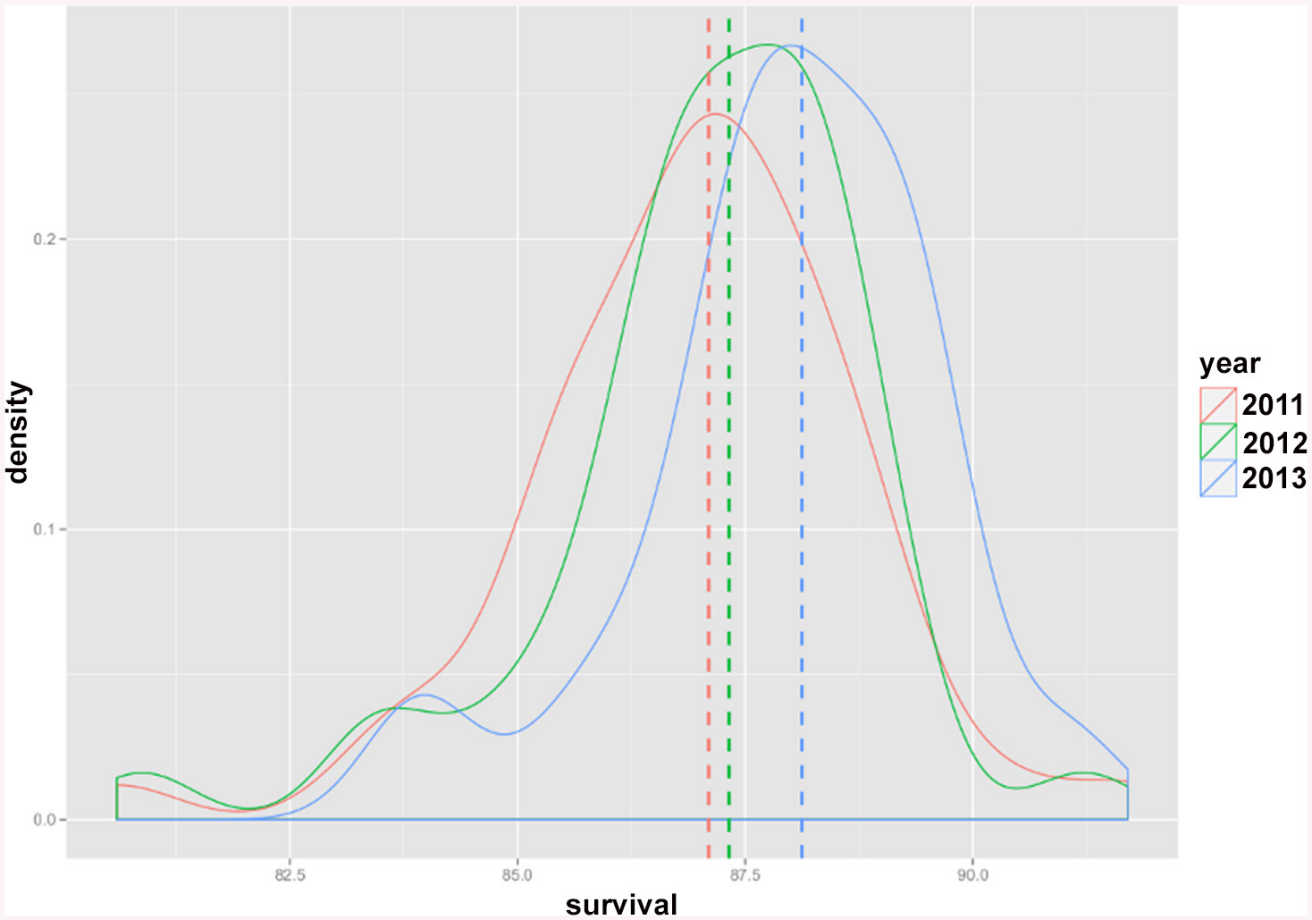
Distribution of estimated risk-adjusted 30-day survival probabilities for AMI diagnosis specific indicator across hospitals. Distribution plots for the risk-adjusted survival probabilities are colored in red, green and blue for the years 2011, 2012 and 2013 respectively. The dotted lines show the reference value for each year.

**Fig 4:**
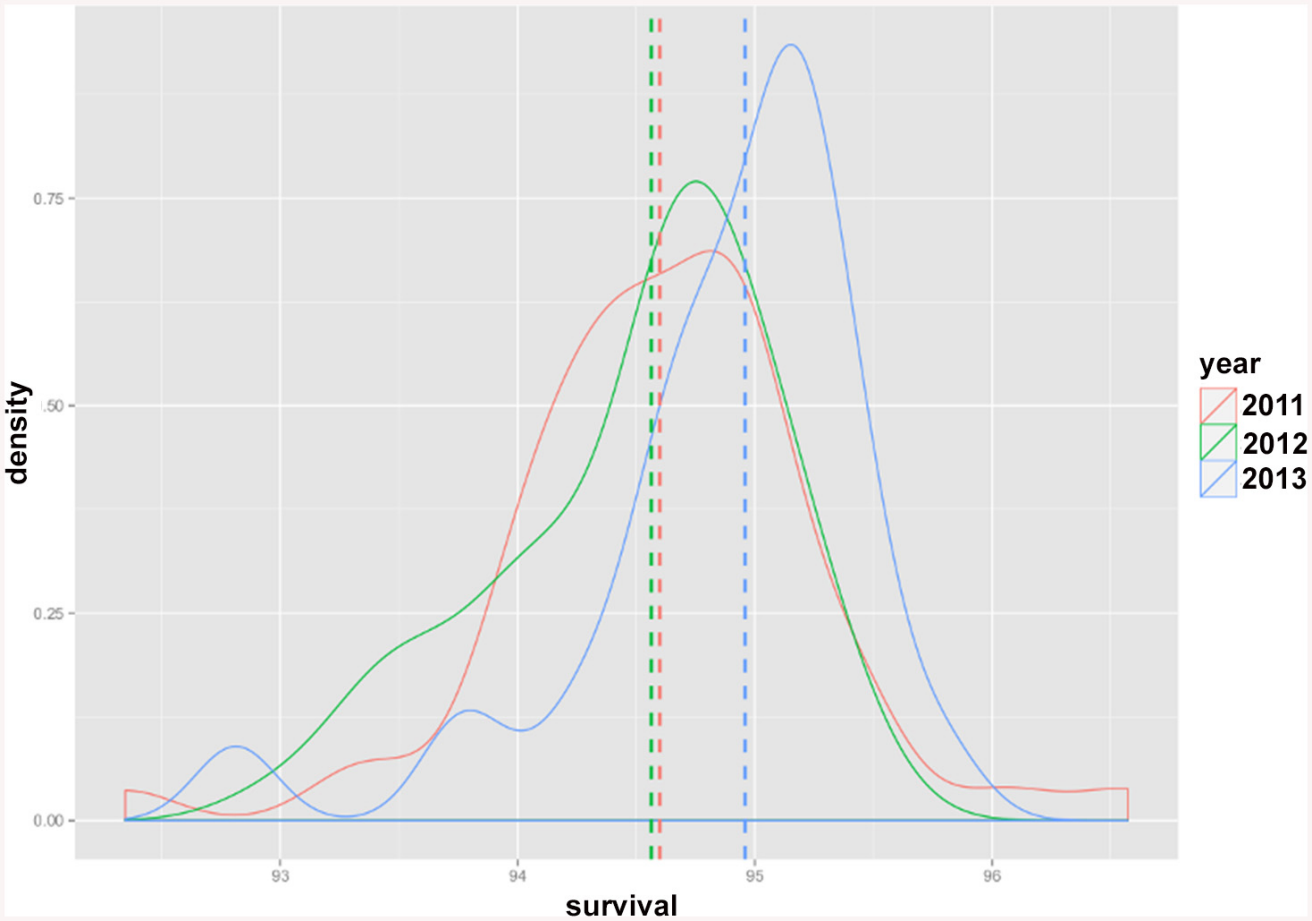
Distribution of estimated risk-adjusted 30-day survival probabilities for the hospital-wide indicator across hospitals. Distribution plots for the risk-adjusted survival probabilities are colored in red, green and blue for the years 2011, 2012 and 2013 respectively. The dotted lines show the reference value for each year.

The Norwegian Knowledge Centre for the Health Services has published survival indicators annually since 2011 at hospital, hospital trust and regional health authority level [10–12]. The results are summarized in Tables 3–5. Total number of hospitals, hospital trusts and regional health authorities as well as the number of those with significantly higher/lower survival (compare to the reference) for each indicator category are given in the above mentioned tables. The highest, lowest and the reference for the risk-adjusted estimated survival probabilities calculated for the hospitals over the last three years are given in Table 6. Interestingly, it may be noticed that variation in estimated risk-adjusted survival indicators i.e. the difference between the highest and lowest estimated survival probabilities for each year, has shown a decreasing trend over time (although not statistically tested). This observed decreasing trend is visualised in Figs 3 and 4, where the distribution of estimated risk-adjusted 30-day survival probabilities for AMI (as an example of the diagnosis specific indicators) and hospital-wide indicator are plotted. The reference values for these two survival indicators show an increasing trend over time (see Figs 3 and 4), indicating increased survival over time.

**Table 3:** Number of hospitals included in the analysis and number of hospitals with significantly higher/lower survival than the reference.

	2011			2012			2013		
Indicator categories	Total	Higher survival	Lower survival	Total	Higher survival	Lower survival	Total	Higher survival	Lower survival
**AMI**	51	4	4	42	7	4	47	3	4
**Hip fracture**	46	1	3	40	0	0	44	0	0
**Stroke**	52	1	5	42	1	1	47	0	0
**Hospital-wide**	52	6	7	51	5	8	51	2	8

**Table 4:** Number of hospital trusts included in the analysis and number of hospital trusts with significantly higher/lower survival than the reference.

	2011			2012			2013		
Indicator categories	Total	Higher survival	Lower survival	Total	Higher survival	Lower survival	Total	Higher survival	Lower survival
**AMI**	22	2	2	22	0	1	23	1	0
**Hip fracture**	21	2	5	21	0	0	21	0	0
**Stroke**	22	3	2	22	0	0	22	0	1
**Hospital-wide**	22	4	4	22	2	5	22	5	5

**Table 5:** Number of regional health authorities with significantly higher/lower survival than the reference.

	2011		2012		2013	
Indicator categories	Higher survival	Lower survival	Higher survival	Lower survival	Higher survival	Lower survival
**AMI**	0	0	0	0	0	0
**Hip fracture**	0	0	1	1	1	1
**Stroke**	0	0	0	1	0	1
**Hospital-wide**	1	2	1	1	0	1

Total number of regional health authorities in Norway is four.

**Table 6:** Risk-adjusted estimated survival probabilities for the 30-day survival indicators (maximum, minimum and reference value) across hospitals

Indicator categories		2011	2012	2013
**AMI**	Highest survival	91.7	91.2	91.2
	Reference value	87.1	87.3	88.1
	Lowest survival	80.6	80.9	83.8
**Hip fracture**	Highest survival	93.2	92.7	93.3
	Reference value	91.6	91.1	91.4
	Lowest survival	89.3	89.9	90.4
**Stroke**	Highest survival	89.3	89.2	88.7
	Reference value	86.5	86.5	86.7
	Lowest survival	82.5	83.9	84.5
**Hospital-wide**	Highest survival	96.6	95.4	95.8
	Reference value	94.6	94.6	95.0
	Lowest survival	92.3	93.0	92.8

## Discussion

A quality indicator, as the name implies, only gives an *indication* of quality, it is not a *direct* measure of quality. Small hospitals that do not deviate significantly might have a too small sample size to be identified in the statistical testing. Another important issue to have in mind is the fact that hospitals that receive patients in the most acute and critical stage may have lower survival rates than hospitals that receive patients who are transferred or are in a more stable phase. This may especially be considered for AMI, where about 50% of patients are transferred.

Estimation of the survival indicators fulfils its purpose if the indicators are actively monitored by the hospital trusts and regional health authorities in order to improve their quality of health care. Since the first public reporting in 2011, several hospitals and hospital trusts in Norway have initiated quality improvement projects, and some of the hospitals have improved the survival over the last four years. This is described in a separate paper, where a before-after study is reported [30].

Several methods for calculating 30-day mortality as a quality indicator have been suggested [29, 31]. A commonly used approach is to calculate the hospital standardized mortality ratio (HSMR), i.e. the ratio of observed versus expected number of deaths [26]. Estimates for the case-mix effects are obtained from a reference population and therefore hospitals may be compared indirectly. Our choice of method compares the hospitals within the same logistic regression model and provides direct comparison of the hospitals [31]. Comparing a few hospitals where some hospitals have very low or very high mortality, the outlier hospitals may highly influence the hospital mean. Therefore, we believe that the trimmed mean is preferable to the common deviance from mean testing, by using a direct comparison approach.

On the other hand, our in-house developed script is specifically designed to handle both PAS data and data from the National Registry. Strengths of our script compared to the other quality indicator software available today, includes but is not limited to: computing survival rather than mortality, assigning hospital weights, pre-processing of the data received, constructing episodes of care, updating the database annually, generating patient population for each indicator with respect to inclusion/exclusion criteria, and running the statistical analyses.

### Limitations

The estimated indicators may only be as accurate as the underlying PAS data. Hospital trusts in Norway are required to document the treatment given to their patients according to a defined coding system for activity based financing based on Diagnosis Related Groups. The Norwegian version of ICD-10 defines the primary diagnosis as the diagnosis most responsible for the hospital care given. This will often represent the greatest length of stay or the greatest consumption of hospital resources and may not necessarily be the reason for the patient’s admission. For example, a hip fracture case may be coded with pneumonia as the main diagnosis, if this complication occurs and requires considerable resources and prolongation of the patient’s length of stay.

For AMI and stroke, severity of the diseases could also affect the estimated indicators. Information regarding the severity currently does not exist in the PAS data and therefore we were not able to consider it in our data modelling. We are planning to retrieve this information and include it in the modelling as an adjustment factor (the National Cardiovascular Disease registry collects this information).

## Conclusions

The methods that NOKC employs for the calculation of four 30-day survival indicators is described in this technical article. Our methods have been tested, employed and revised over the past 10 years [16]. The methods described in this article are the ones that are currently in use. NOKC aims to evaluate alternative approaches for calculating the hospital weights, among other methodological aspects. As our methods develop, the plan is to update this technical article accordingly.

## Softwares

Implementation of the protocol described in this paper was done by means of in-house-written and standard R routines. All data pre-processing and statistical modelling are performed in R version 3.0.3. Baysian shrinkage analysis is performed using JAGS.

## Supporting Information

S1 Appendix Vague Diagnoses. (DOCX)

S2 Appendix Clinical Classifications Software (CCS) categories for all-cause mortality. (DOCX)
