# Supplementary material for: 30-Day Survival Probabilities as a Quality Indicator for Norwegian Hospitals: Data Management and Analysis
Source: PLoS One. 2015 Sep 9;10(9):e0136547. doi: 10.1371/journal.pone.0136547 (PMC4564217; doi:10.1371/journal.pone.0136547)
Supplement: S1 Appendix — (DOCX) [file pone.0136547.s001.docx]

# S1 Appendix. Vague Diagnoses.

A list of the ICD-10 diagnosis codes^[[1]](#footnote-1)^ that are considered as vague diagnoses (in the current article) is given in the following table.

**Table. Vague diagnoses.**

| **ICD-10 diagnosis codes** | **Description** | **ICD-10 diagnosis codes** | **Description** |
| --- | --- | --- | --- |
| **R00.x-R09.x** | Symptoms and signs involving the circulatory and respiratory systems | **Z00.x-Z13.x** | Persons encountering health services for examination and investigation |
| **R10.x-R19.x** | Symptoms and signs involving the digestive system and abdomen | **Z20.x-Z29.x** | Persons with potential health hazards related to communicable diseases |
| **R20.x-R23.x** | Symptoms and signs involving the skin and subcutaneous tissue | **Z30.x-Z39.x** | Persons encountering health services in circumstances related to reproduction |
| **R25.x-R29.x** | Symptoms and signs involving the nervous and musculoskeletal systems | **Excluding**  **Z30.1**  **Z30.2**  **Z31.0**  **Z31.1**  **Z31.2**  **Z31.3**  **Z37.x**  **Z38.x** | Insertion of (intrauterine) contraceptive device  Sterilization  Tuboplasty or vasoplasty after previous sterilization  Artificial insemination  In vitro fertilization  Other assisted fertilization methods  Outcome of delivery  Liveborn infants according to place of birth |
| **R30.x-R39.x** | Symptoms and signs involving the urinary system |  |  |
| **R40.x-R46.x** | Symptoms and signs involving cognition, perception, emotional state and behaviour |  |  |
| **R47.x-R49.x** | Symptoms and signs involving speech and voice |  |  |
| **R50.x-R69.x**  **Excluding**  **R57.x**  **R65.x** | General symptoms and signs  Shock, not elsewhere classified  Systemic Inflammatory Response Syndrome [SIRS] |  |  |
| **R70.x-R79.x** | Abnormal findings on examination of blood, without diagnosis | **Z43.x-Z54.x** | Persons encountering health services for specific procedures and health care |
| **R80.x-R82.x** | Abnormal findings on examination of urine, without diagnosis | **Excluding**  **Z49.x**  **Z50.x**  **Z51.x** | Care involving dialysis  Care involving use of rehabilitation procedures  Other medical care |
| **R83.x-R89.x** | Abnormal findings on examination of other body fluids, substances and tissues, without diagnosis | **Z55.x-Z65.x** | Persons with potential health hazards related to socioeconomic and psychosocial circumstances |
| **R90.x-R94.x** | Abnormal findings on diagnostic imaging and in function studies, without diagnosis | **Z70.x-Z76.x** | Persons encountering health services in other circumstances |
| **V01.x-Y98.x** | External causes of morbidity and mortality | **Z80.x-Z99.x** | Persons with potential health hazards related to family and personal history and certain conditions influencing health status |

1. Diagnosis codes are the ICD-10 diagnosis codes from the Norwegian version of ICD-10 [18]. In this technical note, however, the corresponding English description of the codes are taken from the original WHO version of ICD-10 [19]. Moreover, wherever “x” is written in a diagnosis code, it may be replaced by nothing or anything i.e. an empty string or any number may replace “x”. [↑](#footnote-ref-1)
