# Supplementary material for: 30-Day Survival Probabilities as a Quality Indicator for Norwegian Hospitals: Data Management and Analysis
Source: PLoS One. 2015 Sep 9;10(9):e0136547. doi: 10.1371/journal.pone.0136547 (PMC4564217; doi:10.1371/journal.pone.0136547)
Supplement: S2 Appendix — (DOCX) [file pone.0136547.s002.docx]

# S2 Appendix. Clinical Classifications Software (CCS) categories for all-cause mortality

Clinical Classifications Software (CCS) [21-22] provides a way to classify ICD-10 diagnosis codes into a limited number of categories in order to identify populations for disease- or procedure-specific studies or to develop statistical reports. Further information on the CCS system is available at <http://www.ahrq.gov/research/data/hcup/icd10usrgd.html>. Diagnosis codes that are used are the ICD-10 diagnosis codes from the Norwegian version of ICD-10.

**Diagnostic derivation for Norway**

Previously a list of 44 CCS categories, contributing to 80% of 30-day mortality after hospital admissions in Norway, was used for the calculation of all-cause 30-day survival indicator [9-11]. Recently, we have developed two updated versions of the CCS list. The original list was based on data from 1999-2009, whereas the two new lists are based on data from 2002-2013. The two updated lists represent 80% and 95% of 30-day mortality and contain 42 and 99 CCS categories, respectively. The CCS categories in each of the three lists and corresponding mortality for 2002-2013 data, are given in Table 1. The procedure for choosing the lists of CCS categories is as the following:

**Step 1.** CCS categories are assigned to each episode of care based on the main diagnosis (i.e. the first non-vague primary diagnosis), with the following exceptions:

- CCS 2 - Septicemia (except in labor): Defined by secondary diagnoses, except for CCS categories 224 (Other perinatal conditions) and 195 (Other Complications of birth, puerperium Affecting management of mother).
- CCS 42 - Secondary malignancies: Defined by metastatic cancer as the primary diagnosis, alternatively, as a secondary diagnosis when cancer is stated as the primary diagnosis.
- CCS 45 - Maintenance chemotherapy; radiotherapy. Defined by primary or secondary diagnoses.

**Step 2.** Episodes of care for patients who died within 30 days of admission are identified.

**Step 3.** The data are grouped into the CCS categories, and sorted according to the number of deaths in each group (ascending). Cumulative percentage of the deaths in each group (for 2002-2013) are given in Table 1.

**Step 4.** The top 80% (lists I and II) or 95% (list III) of the CCS diagnosis categories were then chosen, accounting for 80% and 95% of 30-day mortality after hospital admissions in Norway, respectively.

**Table 1. Three lists of CCS categories.** The cumulative percentage of deaths and mortality within 30-days are calculated for the data from 2002-2013

| **CCS category number** | **CCS category name** | **Deaths within 30 days (cumulative percentage)** | **30-day mortality (%)** | **CCS List** | | |
| --- | --- | --- | --- | --- | --- | --- |
|  |  |  |  | **I** | **II** | **III** |
| 122 | Pneumonia (except that caused by tuberculosis or sexually transmitted disease) | 10.89 | 8.98 | X | X | X |
| 109 | Acute cerebrovascular disease | 20.22 | 13.53 | X | X | X |
| 100 | Acute myocardial infarction | 28.77 | 11.41 | X | X | X |
| 2 | Septicemia (except in labor) | 33.39 | 16.98 | X | X | X |
| 226 | Fracture of neck of femur (hip) | 37.27 | 6.61 |  | X | X |
| 108 | Congestive heart failure; nonhypertensive | 41.01 | 8.51 | X | X | X |
| 131 | Respiratory failure; insufficiency; arrest (adult) | 44.41 | 17.71 | X | X | X |
| 19 | Cancer of bronchus; lung | 47.67 | 16.84 | X | X | X |
| 42 | Secondary malignancies | 50.15 | 11.01 | X | X | X |
| 107 | Cardiac arrest and ventricular fibrillation | 52.25 | 59.53 | X | X | X |
| 115 | Aortic; peripheral; and visceral artery aneurysms | 54.14 | 13.51 | X | X | X |
| 127 | Chronic obstructive pulmonary disease and bronchiectasis | 55.88 | 3.77 | X | X | X |
| 14 | Cancer of colon | 57.44 | 8.27 | X | X | X |
| 153 | Gastrointestinal hemorrhage | 58.79 | 5.27 | X | X | X |
| 159 | Urinary tract infections | 60.13 | 2.5 | X | X | X |
| 157 | Acute and unspecified renal failure | 61.40 | 12.89 | X | X | X |
| 114 | Peripheral and visceral atherosclerosis | 62.66 | 4.08 | X | X | X |
| 29 | Cancer of prostate | 63.78 | 4.49 | X | X | X |
| 17 | Cancer of pancreas | 64.88 | 19.85 | X | X | X |
| 233 | Intracranial injury | 65.93 | 1.98 | X | X | X |
| 145 | Intestinal obstruction without hernia | 66.96 | 6.81 | X | X | X |
| 103 | Pulmonary heart disease | 67.99 | 7.54 | X | X | X |
| 24 | Cancer of breast | 68.89 | 3.2 | X | X | X |
| 125 | Acute bronchitis | 69.72 | 2.36 | X | X | X |
| 101 | Coronary atherosclerosis and other heart disease | 70.52 | 0.64 | X | X | X |
| 158 | Chronic renal failure | 71.31 | 4.7 | X | X | X |
| 44 | Neoplasms of unspecified nature or uncertain behavior | 72.03 | 2.49 | X | X | X |
| 106 | Cardiac dysrhythmias | 72.74 | 0.7 | X | X | X |
| 231 | Other fractures | 73.41 | 1.94 | X | X | X |
| 15 | Cancer of rectum and anus | 74.06 | 5 | X | X | X |
| 133 | Other lower respiratory disease | 74.69 | 6.35 | X | X | X |
| 59 | Deficiency and other anemia | 75.32 | 2.9 | X | X | X |
| 13 | Cancer of stomach | 75.93 | 13.49 | X | X | X |
| 129 | Aspiration pneumonitis; food/vomitus | 76.53 | 27.26 | X | X | X |
| 55 | Fluid and electrolyte disorders | 77.13 | 4.42 | X | X | X |
| 39 | Leukemias | 77.72 | 7.2 | X | X | X |
| 149 | Biliary tract disease | 78.29 | 1.03 |  | X | X |
| 96 | Heart valve disorders | 78.82 | 2.83 |  | X | X |
| 155 | Other gastrointestinal disorders | 79.35 | 1.53 | X | X | X |
| 68 | Senility and organic mental disorders | 79.84 | 3.16 |  | X | X |
| 32 | Cancer of bladder | 80.33 | 2.42 | X |  | X |
| 151 | Other liver diseases | 80.78 | 6.54 | X |  | X |
| 230 | Fracture of lower limb | 81.21 | 0.79 | X |  | X |
| 38 | Non-Hodgkin`s lymphoma | 81.63 | 5.12 | X |  | X |
| 130 | Pleurisy; pneumothorax; pulmonary collapse | 82.04 | 3.42 |  |  | X |
| 22 | Melanomas of skin | 82.45 | 10.06 | X |  | X |
| 35 | Cancer of brain and nervous system | 82.85 | 6.23 |  |  | X |
| 27 | Cancer of ovary | 83.26 | 6.12 | X |  | X |
| 43 | Malignant neoplasm without specification of site | 83.65 | 26.17 |  |  | X |
| 239 | Superficial injury; contusion | 84.04 | 1.35 |  |  | X |
| 139 | Gastroduodenal ulcer (except hemorrhage) | 84.42 | 4.79 |  |  | X |
| 40 | Multiple myeloma | 84.77 | 5.72 | X |  | X |
| 244 | Other injuries and conditions due to external causes | 85.11 | 2.59 |  |  | X |
| 199 | Chronic ulcer of skin | 85.45 | 6.13 |  |  | X |
| 18 | Cancer of other GI organs; peritoneum | 85.78 | 8.92 | X |  | X |
| 33 | Cancer of kidney and renal pelvis | 86.11 | 5.84 |  |  | X |
| 146 | Diverticulosis and diverticulitis | 86.43 | 1.66 |  |  | X |
| 12 | Cancer of esophagus | 86.75 | 12.4 |  |  | X |
| 150 | Liver disease; alcohol-related | 87.07 | 8.78 |  |  | X |
| 234 | Crushing injury or internal injury | 87.39 | 1.07 |  |  | X |
| 97 | Peri-; endo-; and myocarditis; cardiomyopathy (except that caused by tuberculosis or sexually transmitted disease) | 87.70 | 3.52 |  |  | X |
| 229 | Fracture of upper limb | 88.01 | 0.48 |  |  | X |
| 11 | Cancer of head and neck | 88.31 | 4.04 |  |  | X |
| 163 | Genitourinary symptoms and ill-defined conditions | 88.60 | 1.28 |  |  | X |
| 116 | Aortic and peripheral arterial embolism or thrombosis | 88.89 | 10.01 |  |  | X |
| 197 | Skin and subcutaneous tissue infections | 89.18 | 0.72 |  |  | X |
| 16 | Cancer of liver and intrahepatic bile duct | 89.45 | 17.56 |  |  | X |
| 3 | Bacterial infection; unspecified site | 89.72 | 5.43 |  |  | X |
| 251 | Abdominal pain | 89.98 | 0.29 |  |  | X |
| 83 | Epilepsy; convulsions | 90.23 | 0.5 |  |  | X |
| 237 | Complication of device; implant or graft | 90.46 | 0.92 |  |  | X |
| 249 | Shock | 90.70 | 48.99 |  |  | X |
| 152 | Pancreatic disorders (not diabetes) | 90.93 | 1.92 |  |  | X |
| 81 | Other hereditary and degenerative nervous system conditions | 91.16 | 1.64 |  |  | X |
| 135 | Intestinal infection | 91.38 | 0.62 |  |  | X |
| 85 | Coma; stupor; and brain damage | 91.59 | 15.79 |  |  | X |
| 41 | Cancer; other and unspecified primary | 91.80 | 9.1 |  |  | X |
| 25 | Cancer of uterus | 91.99 | 3.22 |  |  | X |
| 118 | Phlebitis; thrombophlebitis and thromboembolism | 92.18 | 1.21 |  |  | X |
| 143 | Abdominal hernia | 92.36 | 0.56 |  |  | X |
| 238 | Complications of surgical procedures or medical care | 92.55 | 0.73 |  |  | X |
| 113 | Late effects of cerebrovascular disease | 92.73 | 2.35 |  |  | X |
| 148 | Peritonitis and intestinal abscess | 92.90 | 9.88 |  |  | X |
| 138 | Esophageal disorders | 93.07 | 0.78 |  |  | X |
| 50 | Diabetes mellitus with complications | 93.23 | 1.2 |  |  | X |
| 105 | Conduction disorders | 93.39 | 1.68 |  |  | X |
| 260 | E Codes: All (external causes of injury and poisoning) | 93.55 | 0.73 |  |  | X |
| 117 | Other circulatory disease | 93.70 | 1.11 |  |  | X |
| 134 | Other upper respiratory disease | 93.85 | 0.41 |  |  | X |
| 95 | Other nervous system disorders | 94.00 | 0.19 |  |  | X |
| 112 | Transient cerebral ischemia | 94.15 | 0.6 |  |  | X |
| 211 | Other connective tissue disease | 94.30 | 0.25 |  |  | X |
| 102 | Nonspecific chest pain | 94.44 | 0.16 |  |  | X |
| 254 | Rehabilitation care; fitting of prostheses; and adjustment of devices | 94.57 | 0.2 |  |  | X |
| 205 | Spondylosis; intervertebral disc disorders; other back problems | 94.71 | 0.17 |  |  | X |
| 20 | Cancer; other respiratory and intrathoracic | 94.84 | 15.19 |  |  | X |
